# Generative Adversarial Networks and Its Applications in Biomedical Informatics

**DOI:** 10.3389/fpubh.2020.00164

**Published:** 2020-05-12

**Authors:** Lan Lan, Lei You, Zeyang Zhang, Zhiwei Fan, Weiling Zhao, Nianyin Zeng, Yidong Chen, Xiaobo Zhou

**Affiliations:** ^1^West China Biomedical Big Data Center, West China Hospital, Sichuan University, Chengdu, China; ^2^Center for Computational Systems Medicine, School of Biomedical Informatics, University of Texas Health Science Center at Houston, Houston, TX, United States; ^3^Department of Computer Science and Technology, College of Electronics and Information Engineering, Tongji University, Shanghai, China; ^4^Department of Epidemiology and Health Statistics, West China School of Public Health and West China Fourth Hospital, Sichuan University, Chengdu, China; ^5^Department of Instrumental and Electrical Engineering, Xiamen University, Fujian, China; ^6^Department of Computer Science and Technology, College of Computer Science, Sichuan University, Chengdu, China

**Keywords:** Generative Adversarial Networks (GAN), generator, discriminator, data augmentation, image conversion, biomedical applications

## Abstract

The basic Generative Adversarial Networks (GAN) model is composed of the input vector, generator, and discriminator. Among them, the generator and discriminator are implicit function expressions, usually implemented by deep neural networks. GAN can learn the generative model of any data distribution through adversarial methods with excellent performance. It has been widely applied to different areas since it was proposed in 2014. In this review, we introduced the origin, specific working principle, and development history of GAN, various applications of GAN in digital image processing, Cycle-GAN, and its application in medical imaging analysis, as well as the latest applications of GAN in medical informatics and bioinformatics.

## Introduction

Generative Adversarial Networks (GAN) was introduced into the field of deep learning by Goodfellow et al. (1). As can be seen from its name, GAN, a form of generative models, is trained in an adversarial setting deep neural network. More specifically, GAN learns the generative model of data distribution through adversarial methods. GAN is the most successful generative model developed in recent years and has become one of the hottest research directions in the field of artificial intelligence. Because of its excellent performance, GAN attracts great attention since it was proposed. It is especially important that GAN can not only be used as a generative model with excellent performance, but also its inspiring adversarial learning idea penetrates deeply into all aspects of deep learning, resulting in a series of new research directions and various applications (2).

The basic function of GAN is to train a generator and discriminator in an adversarial way. Based on different requirements of projects, either a stronger generator or a more sensitive discriminator is designed as the target goal. In this manuscript, we focus on the generation purpose of GAN used in four areas: digital image processing, medical image processing, medical informatics, and its latest applications in omic data. The generation purpose can be further categorized into data simulation (3), data augmentation for small dataset (4), style transformation (5), and gene data simulation (6). The great successful applications of GAN in medical image generation (7, 8) and cell gene imputation (6) motivated us to review the literatures in these four sub areas, rather than just focusing on the digital image processing field. We searched in the top conferences of computer science and Google Scholar with keywords related to GAN. Through screening the literature abstracts by our team in digital image processing, medical imaging analysis, medical informatics, and bioinformatics, respectively, the literature that was very relevant to our subject was retained for full text reading. The contents of these eligible literatures are summarized below.

## A Brief Overview of GAN

### Origin of GAN

In general, deep learning models can be divided into discriminant models and generative models (9). In the perspective of the probability and statistical theory, a discriminant model is a method of modeling the relationship between unknown data *y* and known data *x*. A generating model refers to a model that can randomly generate observations, especially under the condition of given some implicit parameters (10). Due to the invention of algorithms such as Back Propagation (BP) and Dropout, the discriminant model has been evolved rapidly. The development of the generative model is lagged due to the difficulty of modeling, though the generative model has a pivotal role in the history of machine learning. When processing large amounts of data, such as images, speech, text, genomics, etc., the generative models can help us simulate the distribution of these high-dimensional data. It will be beneficial for many applications, such as super-resolution, data augmentation, image and medical image conversion, caption generation, electronic health records data generation, biomedical data generation, data imputation, and other ill-posed problems (11–15).

Likelihood describes the probability of the event under different conditions when the results are known (16). Sometimes we may not know the distribution function, but we know the observed data. Therefore, the maximum likelihood estimation is applied to evaluate model parameters using the observed data. Traditional generative models such as Restricted Boltzmann Machine (RBM) (17, 18), Gaussian Mixture Model (GMM) (19), Naive Bayes Model (NBM) (20), Hidden Markov Model (HMM) (20) and so on, are mostly based on maximum likelihood estimate. However, while the explicitly defined probability density function brings computational tractability, maximum likelihood estimation may not represent the complexity of the actual data distribution and cannot learn the high-dimensional data distributions. The majority of generative models require the utilization of Markov chains. GAN uses latent codes to express latent dimensions, control data implicit relationships, etc. and does not require Markov chains (21). Adversarial networks can represent very sharp, even degenerate distributions, while Markov chain-based approaches require somewhat ambiguous distributions so that the chains can be mixed between patterns. Various types of loss functions can be integrated into GAN models. This allows different types of loss functions to be designed for different tasks, all of which can be learned and optimized under the GAN framework. GAN is also a nonparametric modeling method and does not require an approximate distribution of training data to be defined in advance. When probability density is not computable, some traditional generative models that rely on the statistical interpretation of data cannot be used for learning and application. But GAN can still be used in such cases.

### Specific Principles of GAN

In this section, we will introduce the architecture and specific principles of GAN. Basic GAN model is composed of an input vector, a generator, and a discriminator. The generator and discriminator are implicit function expressions, usually implemented by deep neural networks (22).

We use abstract mathematical language to explain the basic principles of the GAN. The fixed distribution *P*_*data*_(*x*) is usually calculated based on the assumption that the data distribution for the training sample *x* is *P*_*data*_. However, this distribution is difficult to be determined. The traditional methods assume that the distribution *P*_*data*_(*x*) obeys a Gaussian mixture distribution and uses the maximum likelihood as the solution. However, when the model is complicated, it is often unable to calculate and the resulting performance is limited (23). This is due to the limited expression ability of the Gaussian distribution itself. Thus, neural networks were proposed to define the distribution *P*_*g*_(*x*). The generator is a neural network with parameter θ^*g*^. It collects the random variable z from the prior distribution and maps it to the pseudo-sample distribution through the neural network, that is, the generated data is recorded as *G*(*z*) and the data distribution is recorded as *P*_*g*_(*z*). The input *z* usually uses Gaussian noise, which is a random variable or a random variable in the potential space. According to θ^g^, a simple input distribution can be used to generate various complex distributions. The *P*_*g*_(*x*) generated by the generator and the real image distribution *P*_*data*_(*x*) should be as similar as possible (24). So, for the generator, the target is to find a G^*^ as shown below.

(1) $$\begin{array}{l} G^{*}=\underset{G}{arg\;min\;Div}(P_{g},P_{data}) \end{array}$$

Then the next question is how to calculate the difference between the two distributions. If the form of *P*_*data*_(*x*) and *P*_*g*_(*x*) is known, it can be calculated to make *P*_*data*_(*x*) and *P*_*g*_(*x*) get close. Although we don't know the specific distribution, we can sample from it. So, GAN proposed a very magical way, discriminator, to calculate the difference between the two distributions. The discriminator was defined by the original GAN as a binary classifier (25) with θ^*d*^. During training, when the input is a real sample *x*, the output of discriminator should be 1, otherwise, the output goes to 0. For defining discriminator, Goodfellow et al. (1) used binary cross entropy function, which is commonly used for binary classification problems.

(2) $$\begin{array}{l} \text{Loss}=-\left(y\;\log\left(ŷ\right)+\left(1-y\right)\log\left(1-ŷ\right)\right) \end{array}$$

Where ŷ is the probability that the model prediction sample is a positive example, and *y* is the sample label. If the sample belongs to a positive example, the value is 1; otherwise, the value is 0. A specific sample may come either from the real distribution or the generated distribution. The positive and negative cases are substituted into *P*_*data*_ and *P*_*g*_, respectively. The whole object function for discriminator is:

(3) $$\begin{array}{l} \text{V}\left(\text{G},\text{D}\right)=E_{x\sim P_{data}}\left[\log D\left(x\right)\right]+E_{x\sim P_{g}}\left[\log\left(1-D\left(x\right)\right)\right] \end{array}$$

By merging Equation (1) into (3), the objective function of the basic GAN is defined by Equation (4):

(4) $$\begin{array}{l} \begin{array}{l} \underset{G}{\min}\underset{D}{\;\max}\;V\left(G,D\right)=\underset{G}{\;\min}\underset{D}{\;\max}\;E_{x\sim P_{data}}\left[\log D\left(x\right)\right]\\ \;+E_{z\sim P_{z}}[\log(1-D(G\left(z\right)))] \end{array} \end{array}$$

By optimizing this objective function, we can get a GAN model. GAN's training can be regarded as a min–max optimization process. The generator wants to deceive the discriminator, so it tries to maximize discriminator's output when a fake sample is presented to the discriminator. Instead, the discriminator attempts to distinguish the difference between real and false samples. Consequently, discriminator tries to maximize *V* (*G, D*) while generator tries to minimize *V* (*G, D*), thus forming the minimax relationship. During the training of GAN, the parameters of *G* (θ^*g*^) and *D* (θ^*d*^) are continuously updated. When the generator is undergoing training, the parameters of the discriminator are fixed. The data generated by the generator is marked as fake and input into the discriminator. The error is calculated between the output of the discriminator *D* (*G*(*z*)) and the sample label, and the parameters of generator are updated using the error of BP algorithm. When the discriminator is undergoing training, the parameters of the generator being fixed. Discriminator gets positive sample *x* from the real data set, and the generator generates a negative sample *G*(*z*). The output of the discriminator and sample labels are used to calculate the error. Finally, the parameters of the discriminator are updated by the error of BP algorithm.

Ideally, the generator and discriminator are in equilibrium when *P*_*data*_(*x*) = *P*_*g*_(*x*). When the generator is fixed, we can take the derivative of *V* (*D, G*) to find the optimal discriminator D^*^(*x*), as shown in the Equation (5).

(5) $$\begin{array}{l} D^{*}\left(x\right)=\frac{P_{g}\left(x\right)}{P_{g}\left(x\right)+P_{data}\left(x\right)} \end{array}$$

By substituting the optimal discriminator in the Equation (3).

(6) $$\begin{array}{l} \underset{D}{\max}\;V(G,D)=-2\log2+2JSD(P_{data}(x)∥P_{g}(x)) \end{array}$$

The objective function can be further calculated as optimizing the JS divergence of *P*_*data*_(*x*) and *P*_*g*_(*x*) under the optimal discriminator (26).

### Development History of GAN

GAN is an excellent generative model. However, the original GAN model has many problems, such as the vanishing gradient, difficulty in training, and poor diversity (27). Many efforts have been devoted to obtaining better GANs through different optimization methods. Therefore, since 2014, theories and articles related to GAN have emerged in an endless stream, and many new GANs-based models have been proposed to improve the stability and quality of the generated results (28).

A number of review articles have summarized and classified the current GAN-related models (22, 24, 29). Creswell et al. (22) classified the evolution of GAN models from the aspects of architectural development and loss function improvement. Hong et al. (29) summarized the development of GAN models from the aspects of theoretical analysis, supervised, unsupervised, and common problems. Guo et al. (24) focused on the improvement of the model structure, the expansion of the theory, the novel application and so on. We will introduce several common improvements of GAN here.

#### Conditional Generative Adversarial Networks (CGAN)

CGAN is an improved GAN model proposed by Mirza et al. (30). Unlike the original GAN, CGAN uses a supervised approach increasing controllability of generated results. CGAN takes the random noise *z* and the category label *c* as inputs of the generator and the generated sample/real sample and category label as inputs of the discriminator to learn the correlation between labels and images. By introducing a conditional variable *y* into the modeling and adding conditions to the model with additional information y, the data generation process can be guided.

#### Deep Convolutional Generative Adversarial Networks (DCGAN)

One year after the first GAN paper was published, researchers found that the GAN model was unstable and required a lot of training skills. In 2015, Radford et al. (31) proposed an upgraded version of the GAN architecture, named DCGAN. The authors of DCGAN improved the architecture of the original GAN with deep convolutional networks (CNNs). So far, DCGAN's network structure is still widely used and is the hottest GAN architecture and a milestone in the history of GAN. Compared with the original GAN, DCGAN almost completely uses the convolution layer instead of the fully connected layer. The discriminator is almost symmetric with the generator. The entire network does not have pooling layers and up-sampling layers. DCGAN also used Batch Normalization algorithm to solve the problem of vanishing gradient.

#### f-GAN

The objective function of the original GAN can be seen as minimizing the JS divergence between two distributions. In fact, there are many ways to measure the distance between two distributions, and JS divergence is just one of them. Defining different distance metrics can result in different objective functions. Nowozin et al. (32) applied *f*-divergence to GAN (*f*-GAN) for training generative neural samplers. The *f*-divergence is a function *D*_*f*_(*P*∥*Q*) that measures the difference between two probability distributions *P* and *Q*. Under the framework of *f*-divergence, *f*-GAN generalizes various divergences so that the corresponding GAN target can be derived for a specific divergence. Many common divergences (33), such as KL-divergence, Hellinger distance, and total variation distance, are the special cases of *f*-divergence, coinciding with a particular choice of *f* . Many improvements in GAN training stability are achieved by using different distance metrics between distributions, such as Energy-based GAN (EBGAN) (34), Least Squares GAN (LSGAN) (35), etc.

#### Wasserstein Generative Adversarial Networks (WGAN)

WGAN mainly improved GAN from the perspective of the loss function. WGAN theoretically explained the reason for the instability of GAN training, that is, cross entropy (JS divergence) is not suitable for measuring the distance between distributions with disjoint parts. Therefore, WGAN proposed a new distance measurement method, Earth Moving Distance, also known as Wasserstein distance or optimal transmission distance, which refers to the minimum transmission quality that converts the probability distribution *q* to *p* (probability density is called probability quality in discrete cases) (26, 36). The superiority of Wasserstein distance compared to KL divergence and JS divergence is that even if two distributions do not overlap, Wasserstein distance can still reflect their distance. The theoretical derivation and interpretation of WGAN are quite complicated. The authors of WGAN (26) pointed out that the use of Wasserstein distances needs to satisfy a strong continuity condition, i.e. Lipchitz continuity.

In short, GAN still has many unresolved problems and can be further improved in various aspects.

## Application of GAN in Image Processing

**Figure 1:**
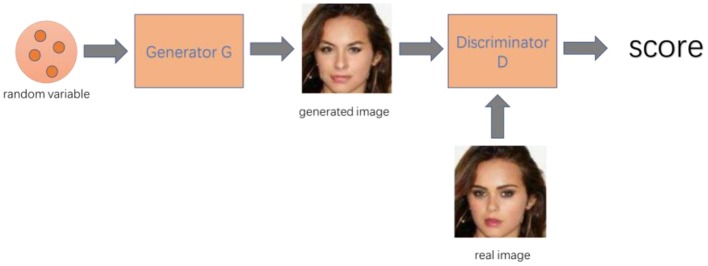
Digital image diagram of GAN.

GAN is widely used in virtual image generation (Table 1). Whether it is a face image, a room scene image, a real image (37) such as a flower or an animal, or an artistic creation image such as an anime character (39), it can be learned using GAN to generate new similar images (Figure 1). GAN is fully utilizing its unique advantages and has evolved from the original GAN to the progressively growing GAN (proGAN). Its imaging generation capability has been greatly improved from 32 × 32 resolution to 2K true and false HD resolution (37).

**Table 1:** Literatures for the application of GAN in image processing.

**References**	**Model**	**Public dataset**
Mirza and Osindero (30)	CGAN	MNIST
Radford et al. (31)	DCGAN	LSUN, IMAGENET-1K
Nowozin et al. (32)	*f*-GAN	MNIST Digits, LSUN
Zhao et al. (34)	EBGAN	MNIST digit, LSUN, CelebA, ImageNet
Arjovsky et al. (26)	WGAN	LSUN-Bedrooms
Karras et al. (37)	proGAN	CelebA, LSUN
Ledig et al. (12)	SRGAN	Set5, Set14, BSD100
Pathak et al. (38)	Context encoder	Paris Street View, ImageNet

Image super-resolution task (SR) is to generate high-resolution images from low-resolution images. Image super-resolution algorithms are important in areas such as video surveillance, medical diagnostics, and remote sensing applications. Super-resolution problem is actually an ill-posed problem because the lost high-frequency details are difficult to recover during the resolution of the image. Traditional methods are generally interpolated, and interpolation inevitably creates blurring. However, GAN can learn the distribution of high-resolution images to a certain extent, so that high-quality images with better quality can be generated. CNN has also achieved very good results in single-frame super-resolution reconstruction and can achieve a higher peak signal-to-noise ratio (PSNR) (40, 41). However, most of them use MSE as the objective function. Although a higher peak signal-to-noise ratio can be achieved using MSE, when the image down-sampling is higher (four times), the reconstructed picture will be too smooth and lose details. Thus, in 2016, Super Resolution GAN (SRGAN) was proposed by Ledig and others of Twitter. SRGAN was the first to propose the application of GAN to super-resolution reconstruction (12). The generated model of SRGAN takes a blurred low-resolution image as input and outputs a clear image with high resolution. The discriminant model of SRGAN determines whether the input image is a “true high-resolution image” or a high-resolution image converted from a low-resolution image. This greatly simplifies the learning process of the image super-resolution model. Because traditionally conducting an image super-resolution needs to model some high-frequency details, but here the purpose of generating model training is simplified to the confusing discriminant model. Compared to previous results based on deep learning models for image super-resolution such as SRResNet, etc., GAN's images can provide more details.

Image inpainting refers to the process of reconstructing missing or damaged parts of images and videos. It involves in image editing and image generation and is a process of artificially filling a region where information on the image is missing according to certain rules. Conventional image inpainting methods typically utilize undamaged image information to estimate missing portions and autofill the missing parts. Therefore, a structure-based partial differential equation (PDE) image restoration algorithms were proposed. The repair process of these models is similar to the diffusion phenomenon of physics, and the key and the difficulty lies in how to build a diffusion model. When the image damage area is large, the repair effect plummets. Texture-based image inpainting algorithms emerged as the times require, and the effect based on partial differential equations was improved to some extent. Because the traditional image inpainting algorithms depend on the structure or texture information of the image, they cannot meet basic repair requirements, and often cannot achieve satisfactory results, when the image semantic information is missing. Deep learning has strong learning ability and can learn advanced features from images, so the inpainting problem can be solved with such features (42–44). Image inpainting is a problem between image editing and image generation, so using the GAN model can solve this problem well. The solution for using GAN is to input an image with missing part to the generator. The generator will use this missing input image to generate a new complete image. The discriminator will learn to judge whether this image is realistic enough and feedback to the generator. Through continuous training to optimize, the generator can finally generate a complete image that is sufficiently realistic. Then the inpainting was finished. Context encoder (38) is a pioneering work of deep learning in the field of image inpainting. Pathak et al. (38) trains an encoder-decoder and combines adversarial network loss to predict the missing portion according to the context pixel and structural semantics of the missing area. The network is able to obtain a reasonable image structure and can quickly and accurately evaluate the repair results (45–47). Because GAN's generator and discriminator can be any form of neural network, different network architectures can be selected for solving different problems. The autoencoder model was used as the generator part in our previous work (3). This is because the autoencoder is also an important generative model. It encodes the input data and generates new data through the decoding operation. It can retain the characteristics of the original input and introduce the newly generated part. Therefore, it can not only keep the undefected part of the inpainting input, but also generate some data for filling in the missing area by using autoencoder. Our discriminator is a simple binary classification discriminant neural network whose input are the generated repaired image and the complete image in the original dataset. By learning to distinguish between the two, the generator is prompted to generate results that are more in line with the dataset, thereby completing the inpainting.

## Application of GAN in Medical Image Processing

Multiple factors such as time cost, labor cost, economic cost, etc. make it more difficult for researchers to acquire labeled medical images than normal images. However, there is a great demand for medical images by scholars nowadays. For example, the deep learning-based model can achieve a better performance in the fields of medical image segmentation, classification, registration, etc. than the hand-crafted features when dealing with a large amount of data (48). Traditional image augmentation methods can be used for its purpose. However, the generated images by traditional augmentation methods share a similar distribution with the original ones (49, 50). Those methods are not suitable for the need of generating more incidences among different patients. Accordingly, GAN is used more popular in medical image analysis, such as data augmentation and multi-modality image translations.

Recently, with the development of deep learning algorithms and the growing of labeled image datasets, convolutional neural networks (CNN)-based models (51) have achieved great success in many computer vision tasks, such as object detection (52), semantic segmentation (53), human action recognition (54) and so on.

Since 2014, many CNN-based medical image analysis works (55, 56) have shown great learning possibility when enough images are available for model training. The database like TCGA (57) supplies a large number of images for some common diseases. Since image acquisition and annotation is a time-consuming process, image data for many diseases remains scarce.

There are many deep learning models that are pre-trained on larger image datasets such as ImageNet (58), COCO (59), and so on. Transfer learning (60) uses limited labeled data for supervised training. In the transfer learning tasks, most of the weights of the model keep fixed and only the weights of the last several layers are fine-tuned on the new dataset. In this way, a well pre-trained deep neural network is applied in the medical image analysis.

Transfer learning may still suffer from lack of training images (61). As mentioned above, traditional data augmentation methods can only generate data that share a close distribution with the original ones. If the data set is too small, these methods almost have no effort on the data augmentation. GAN (26) supplies a solution to the lack of data in medical image analysis. In the following section, we will discuss the applications of single GAN and Cycle-GAN in medical image analysis (Table 2).

**Table 2:** Literatures for the application of GAN in medical image processing.

**References**	**Model**	**Public Dataset**
Zhang et al. (62)	PAC-GAN	VIPeR, CUHK03, Market-1501
Dirvanauskas et al. (63)	GAN/medical	Miri TL
Pandey et al. (64)	Two-stage GAN/Medical	Kaggle Data Science bowl's first stage of competition
Frid-Adar et al. (65)	GAN	Private
Chen et al. (66, 67)	Dense GAN	A large publicly accessible brain structural MRI database
Mahapatra and Bozorgtabar (68)	Skip-connection GAN	http://www.eyepacs.com/
Yi and Babyn (69)	SAGAN	National Cancer Imaging Archive
Shitrit and Raviv (70)	GAN	Private
Zhu et al. (5)	Cycle GAN	Cityscapes, Google Maps, CMP Facade Database, UT Zappos50K, ImageNet
Wolterink et al. (71)	Cycle GAN	Private
Hiasa et al. (4)	Cycle GAN	Private
Huo et al. (72)	Cycle GAN	Private
Tanner et al. (73)	Cycle GAN	Private
Zhang et al. (74)	Cycle GAN	Private
Zhang et al. (75)	Cycle GAN	Private

### GAN Used in Medical Image Analysis

Pandey et al. (64) proposed a two-stage strategy to generate nuclei cell images and their corresponding masks based on GAN. In their first stage, a generator is trained to generate the synthesis masks from noise like conventional GAN model does. On their second stage, a conditional GAN utilized real mask and random noises to train a generator for synthesizing images. Finally, they utilized these two generators to generate images and masks from random noise.

Dirvanauskas et al. (63) generated human embryo cell images for three stages (one-cell, two-cell, and four-cell) by a conventional GAN model. All the synthesized images could be used to facilitate the development, training, and evaluation for embryo image processing tasks.

Zhang et al. (62) used GAN-based model to solve the data shortage problems in person re-identification task. Two view images (cross view images) are generated by a conditional GAN from existing original images and skeleton images. After that, these cross-view images are sent into a discriminator for person re-identification.

Frid-Adar et al. (65) used two variations of GAN models to generate synthetic liver lesions. The synthetic images contained the regions of interest (ROI) on abdomen CT images with a resolution of 64 × 64. Experiment results showed that the synthetic data augmentation from these two GAN models improved classification accuracy from 77.5 to 85.0% compared to the classic data augmentation.

Chen et al. (66) proposed a high-resolution MRI (HR MRI) image generation architecture. Instead of generating 2D HR MRIs, the authors generated 3D HR MRIs to learn 3D structures of MRI volumetric images. However, 3D networks bring more computing requirements. To solve this problem, the authors used 3D dense net-based architecture (67) in the generator. By combining 3D dense net and GAN, synthetic HR MRIs have more local image textures and details.

Mahapatra and Bozorgtabar (68) used local saliency maps and GAN for generating high-resolution retinal images. In addition to the GAN's lost function, they also added local saliency loss from the difference between HR images and low-resolution images in the saliency maps.

Yi and Babyn (69) proposed a deep neural network-based architecture for low dose CT denoizing. The generator-synthesized denoized CT image was sent to a sharpness detection network for comparison with a conventional CT image. This branch contributes a sharpness lost for the GAN objective function.

Shitrit and Raviv (70) applied GAN to accelerate the MRI image generation process. Instead of generating MRI images from existing MRI images, the authors used GAN to generate missing *k*-space samples. Their approach can be used for time-sensitive or resolution-sensitive MRI scan tasks.

Huo et al. (72) proposed a similar GAN architecture for splenomegaly segmentation. Instead of generating images from random noises, the authors used a U-Net (76) based architecture to get a segmented version and a Dice lost function as the discriminator. Isola et al. (77) used a Patch-GAN model as the discriminator for the patches from both the generated images and the ground truth images.

### Cycle-GAN Used in Medical Image Analysis

**Figure 2:**
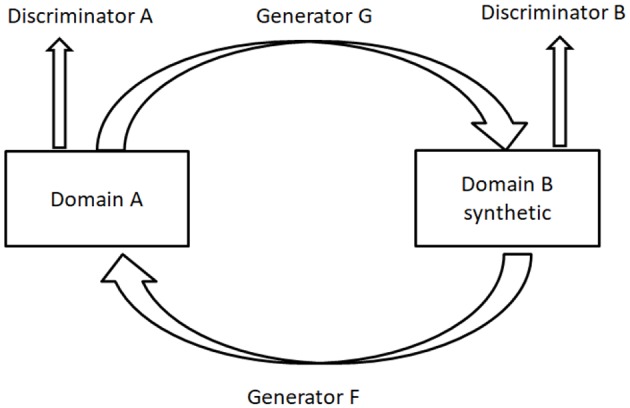
The architecture of Cycle-GAN.

Cycle-GAN (5) is utilized to learn the mapping from a domain image set *X* (or *A* in Figure 2) to another domain image set *Y* (or *B* in Figure 2) when the pairwise alignments between the two domains are unavailable. The forward generator is defined as G and the backward generator as F. The cycle consistency forces *F*(*G*(*x*))≈*x* and *G*(*F*(*y*)) ≈y. *F*°*G* or *G*°*F* works similarly to an auto-encoder (78) for learning the representations of the original images. A similar method was presented by Yi et al. (79).

It is essential that images from two different sources must have shared visual content, such as from wild horses to zebras. Normal image translation may be greatly affected when there is a significant difference between two domains. However, it is more suitable for multi-modality medical image synthesis, such as synthesizing images from Magnetic Resonance Imaging (MRI) to Computed Tomography (CT) (71) for diagnosing some specific diseases.

Wolterink et al. (71) applied Cycle-GAN to CT and MRI images of brain tumors in the radiotherapy treatment planning. Since a limited number of patients had paired CT and MRI images, unpaired images were generated from the paired images by first padding the images into a larger resolution and then cropping them into the same smaller resolution. Their results showed that Cycle-GAN trained with unpaired images outperforms a single GAN trained with paired images.

Hiasa et al. (4) improved the Cycle-GAN architecture by adding the gradient consistency loss with the goal of better edge alignment between the MRI images and CT images. Comparing the generated CT/MRI images with the actual ones, the gradient consistency had improved the synthesis accuracy and segmentation accuracy.

Huo et al. (80) combined Cycle-GAN and segmentation network in an end-to-end manner to take advantage of the complementary information between synthesis and segmentation. The final lost function of their network consisted of the Cycle-GAN lost functions and the segmentation loss function. Compared with the first synthesis and then segmentation method (81), the method from Huo et al. achieved better segmentation results on the spleen and other organs. Their experiments indicate that MRI images with multiple organ labels can be used to generate corresponding segmented CT images. Tanner et al. (73) used Cycle-GAN for MRI-CT deformable image registration of thoracic and abdominal organs. Jin et al. (82) applied Cycle-GAN for CT-MRI image synthesis. A discriminator was added for the real paired CT-MRI images and generated paired CT-MRI images. The combination of paired images and unpaired images achieved the lowest mean absolute error.

Yue et al. (74) proposed a task-driven generative model for X-ray image segmentation. A U-Net-based network (76) was trained supervised on Digitally Reconstructed Radiographs (DRRs) for organs segmentation. Thereafter, a Cycle-GAN was trained for DRRs and X-ray images synthesis. Specifically, the segmentation loss generated by the previously trained segmentation network was added to the cycle of real DRRs to fake X-ray to reconstructed DRRs. In that case, the segmentation results of X-ray images were greatly improved.

Zhang et al. (75) replaced discriminators with segmenters to address shape consistency problem. 3D fully convolution layers were used in the Cycle-GAN network and long-range U-Net network. Experiment results showed that the Cycle-GAN-based synthesis network and segmentation network were mutually beneficial in segmenting cardiovascular volumes.

**Figure 3:**
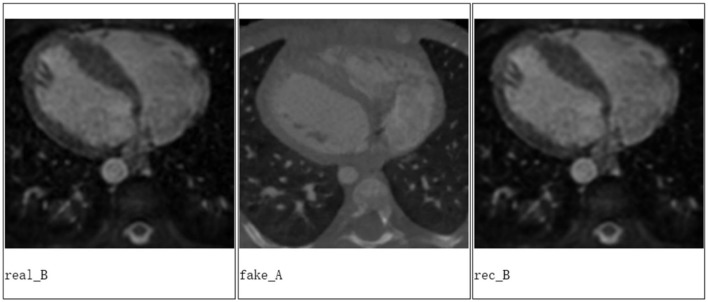
The experiment results from Cycle-GAN, where real B is the real MRI image, fake A is the generated CT image based on the real MRI image, and rec B is the reconstructed MRI image based on the generated CT image of heart for a patient.

We applied Cycle-GAN to achieve a good conversion between the CT and MRI images based on the data from MICCAI Workshop (Figure 3).

## Application of GAN in Medical Informatics

With the development of health informatization, hospital information systems, Internet of Things (IoT)-based health Platform, wearable devices and other platforms have led to the explosive growth of medical data, such as electronic medical records (EMR) (83). The growth in the quantity and quality of medical data has also facilitated the use of scientific research and algorithms in medicine. However, due to data security, especially privacy security, although patient's data can be de-identified, the medical data after de-identification can still be re-identified by some combinations. Because there are barriers between the health information systems, it is very difficult to correlate medical data collected from different media, resulting in less medical data available for scientific research. The application of medical informatics often requires a large number of data to train parameters. The lack of medical data severely limits the application of deep learning algorithms, especially artificial intelligence in the field of medical informatics. Therefore, the development of medical informatics is behind fields such as medical images.

GAN has proven to play an important role in generating images and has shown good performance in generating continuous data. Since the gradient function is required to be differentiable, the traditional GAN cannot generate discrete data. In medical data, the diagnosis of a disease and the severity of the disease are discrete data. Due to the high cost, less labeled, and unbalanced classification medical data are available. Therefore, we explored the application of GAN in generating discrete data based on real medical data and in solving problems such as fewer labels and unbalanced classifications (Table 3).

**Table 3:** Literatures for the application of GAN in medical informatics.

**References**	**Model**	**Public dataset**
Choi et al. (84)	medGAN	PAMF, MIMIC III
Baowaly et al. (85)	medGAN, WGAN-GP, BGAN	MIMIC-III
Yoon et al. (86)	RadialGAN	MAGGIC
Che et al. (87)	ehrGAN	Private
Esteban et al. (88)	RGAN, RCGAN	Philips eICU database
Li et al. (89)	GAN	IQVIA longitudinal prescription (Rx) and medical claims (Dx) database
Guan et al. (90)	mtGAN	Private
Yang et al. (91)	GAN	UCI medical database, Cerebral stroke dataset
Tang et al. (92)	IRGAN	4705 hyperlipidemia questions from the internet
Hassouni et al. (93)	GAN	WISDM

Choi et al. (84) generated synthetic electronic health records (EHR) by using medical Generative Adversarial Networks (medGAN) based on the Sutter Palo Alto Medical Foundation (PAMF) and the Medical Information Mart for Intensive Care (MIMIC-III) datasets. The original GAN cannot be directly used to learn the discrete data of patients. medGAN can handle high-dimensional multi-label discrete variables (binary and count variables such as diagnoses, medications, and procedure codes) by leveraging the autoencoder to overcome the limitation from the original GAN. The autoencoder learned from real patient records and the same decoder in autoencoder was used to construct the discrete output after the generator. The authors obtained impressive results for discrete variables. Baowaly et al. (85) synthesized more realistic EHR than those generated by the medGAN using MIMIC-III, extended MIMIC-III, and Taiwan National Health Insurance Research Database (NHIRD). Two synthetic data generation models, Wasserstein GAN with gradient penalty (WGAN-GP) and boundary-seeking GAN (BGAN), were applied based on the medGAN framework. These two GAN models were named as medWGAN and medBGAN, respectively. The count (the frequency of a specific ICD or procedure of disease) and binary data (presence or absence of a specific ICD code) were created using medGAN, medWGAN, and medBGAN. Their results showed that the two improved GAN models outperformed the medGAN. medBGAN performed best in these three models.

Yoon et al. (86) used the auxiliary datasets, external datasets from related but different hospitals, as the noise for a GAN framework based on the fact that the patient distribution from one hospital will be better matched by the patient distribution from another hospital than by random noise such as Gaussian, enlarging the target dataset effectively. They used 14 studies of MAGGIC to create target datasets and compared the prediction performance between the proposed radialGAN, target-only GAN and benchmarks such as conditional-GAN and starGAN, etc. Integrating datasets from different hospitals by radialGAN can improve the performance of target-specific predictive models.

Che et al. (87) used two longitudinal real clinical datasets of heart failure and diabetes to investigate how well ehrGAN generated EHR as real samples. The structure of the basic prediction model was adopted in the discriminator. Based on the variational contrastive divergence, the generator was altered for semi-supervised learning setting. Data augmentation was performed by semi-supervised learning utilizing ehrGAN to boost risk prediction; thus, generalization capacity and prediction performance were improved.

**Figure 4:**
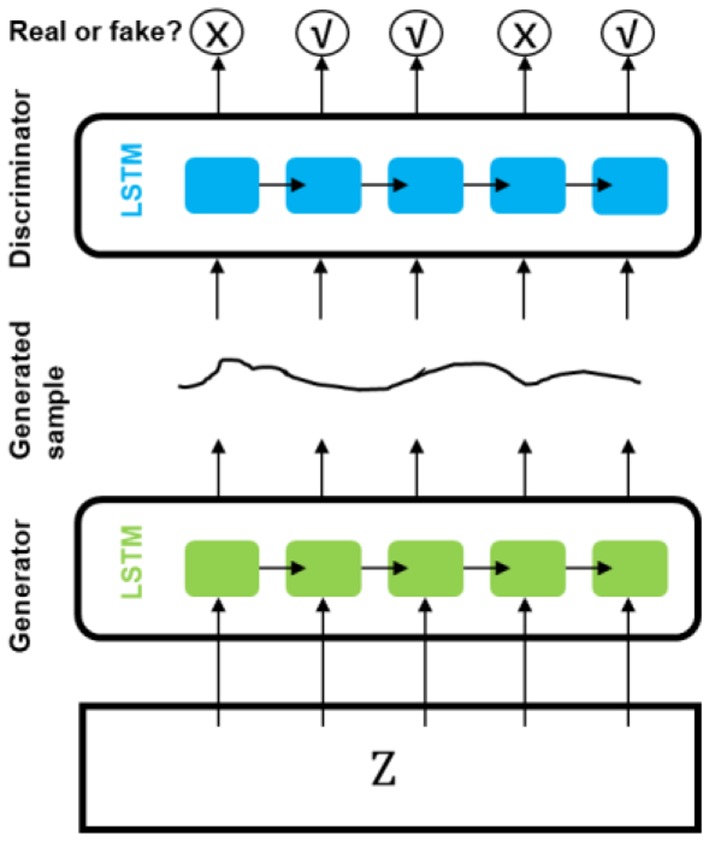
The architecture of LSTM-based GAN in medical informatics.

Esteban et al. (88) presented a recurrent GAN (RGAN) and a recurrent conditional GAN (RCGAN) to generate sequences without/with some conditional inputs. Long Short-Term Memory (LSTM) was selected as the architecture for both discriminator and generator. They predicted whether or not a patient will become “critical” in the near future based on the four most frequently recorded variables measured by bedside monitors from Philips eICU database using a method named “Train on Synthetic, Test on Real” (TSTR). The models trained on the synthetic dataset from LSTM-based GAN (Figure 4) achieved performance at times comparable to that of the real data on the eICU dataset.

Li et al. (89) used GAN to predict if a patient has a rare disease. The log probability of unlabeled data as real data was maximized and added in the objective function of the discriminator based on the IQVIA longitudinal prescription and medical claims database. Compared to the baseline techniques, the prediction accuracy of the semi-supervised learning framework for rare disease detection (precision-recall curves and area under the curve) was 5% higher.

Guan et al. (90) used medical text GAN (mtGAN) to reinforce the electronic medical record texts. mtGAN is a conditional GAN that takes designated disease features as input and generates corresponding EMR text to address privacy issues as well as inadequate and unbalanced and the insufficient and imbalance samples problem.

Yang et al. (91) presented a semi-supervised method in association with GAN to support medical decision making. In their study, GAN generated synthetic data by taking labeled set as input. Labels of the unlabeled set were predicted by two learners. By taking the expanded labeled set as input, GAN was used again to generate the labeled set. Both expanded labeled set and synthetic set were used as the training set to be classified based on the cerebral stroke set collected from IoT-based platform.

Tang et al. (92) proposed a GAN-based method to automatically retrieve patient questions. Supervised deep learning-based approaches were used to determine the similarity between patient questions. Their study showed that fine tuning with GAN can improve performance. Hassouni et al. (93) used GAN with LSTM to generate realistic simulation environments based on the WISDM dataset. Their results showed that the model trained on the data artificially generated by the GAN had similar performance trained on real data.

## Application of GAN in Bioinformatics

As a branch of the life science, bioinformatics is a new multidisciplinary field that understands and organizes information related to biomolecules through a combination of disciplines such as applied mathematics, biology, computer science, and statistics (94). It applies conventional statistics, modern computer science, machine learning, and other modeling algorithms to explore large volumes of biological data, including molecular sequences of DNA, RNA, proteins and metabolites, and other whole genome data. Bioinformatics research and applications include analysis of molecular sequence and genomics data; genome annotation; molecular folding, modeling, and design; building biological networks; analysis of the cellular organization and computational evolutionary biology (95, 96).

**Figure 5:**
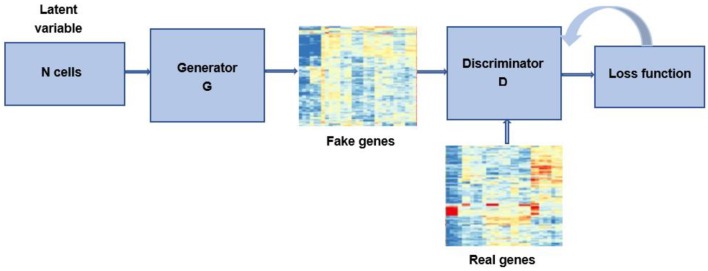
Bioinformatics diagram of GAN.

One of the most important and difficult issues for bioinformatics researchers is the accessibility and availability of large datasets. Though the increased throughput and technological advances have changed the landscape of metagenomics, the cost of sequencing is still relatively high. In addition, since the accessibility of data for research purpose involves many legal and ethical issues, bioinformatics data is highly sensitive (97). The lacking of available biological samples could result in imbalanced datasets, which can lead to over-fitting problems and poor classification performance. Recently, researches have used GAN to generate data samples to overcome these problems (Figure 5). Here, we present some of bioinformatics application cases (Table 4).

**Table 4:** Literatures for the application of GAN in bioinformatics.

**References**	**Model**	**Public dataset**
Ghahramani et al. (98)	Wasserstein-GAN (WGAN) with gradient penalty loss function	GSE90848, GSE67602, GSE99989
Marouf et al. (99)	Conditional single-cell GAN	68,579 PBMCs (healthy donor A)
Xu et al. (6)	Generative adversarial networks for scRNA-seq imputation	GSE65525
Li et al. (100)	GAN	Not applicable
Anand and Huang (101)	GAN	Protein Data Bank
Killoran et al. (102)	GAN	Not applicable
Gupta and Zou (103)	Feedback GAN	Uniprot database
Wang et al. (104)	GAN	GEO, GTEx, 1000 G RNA-Seq expression data

Ghahramani et al. (98) successfully simulated realistic scRNA-seq data using WGAN-GP, covering diverse scRNA-seq datasets of various cell types, including mouse epidermal, mouse neural and human hematopoietic single-cell RNA-seq data spanning from different laboratories and experimental protocols. The performance was evaluated at different checkpoints using *t*-distributed stochastic neighbor embedding (*t*-SNE) and the correlation between real cells and generated cells. As the training steps increased, the generator was capable of producing cells mapping to multiple clusters in the *t*-SNE plot, covering different cell types, cell states, cell origin and experimental batches present in the combined real dataset (98). By using the generative model, the researchers were able to obtain a universal representation of epidermal differentiation and the representation features can be used to predict the effect of cell state perturbations on gene expression at high temporal resolution. The task of the generator was to produce realistic output data from a random latent space vectors *z*. Corresponding latent space vectors of terminally differentiated and undifferentiated cells (*z*_*differentiated*_and *z*_*basal*_) were obtained according to the correlation between the real expression profiles and those generated, and the difference between these vectors was calculated as δ = *z*_*differentiated*_−*z*_*basal*_. Then 1,000 time points were interpolated between the latent space differentiation vectors δ (98), so the dynamics of time-series gene expression over cell differentiation could be explored using the GAN model, which cannot be detected by experiments. By doing so, transiently expressed and subsequently down-regulated genes associated with the process of differentiation can be identified. They also performed a sensitivity analysis of the discriminator network to identify biological state-determining genes. By analyzing those networks, the authors obtained regulatory relationships for inferred genes.

Marouf et al. (99) built a single cell GAN model for scRNA-seq data generation using a Peripheral Blood Mononuclear Cell (PBMC) scRNA-seq dataset with 68,579 cells. A customed Library Size Normalization (LSN) function and Fully-Connected Neural Network with Batch Normalization were used in the scGAN's generator to improve training speed and stability. scGAN was able to model the dependency and correlation of intergenic, which are a hallmark of biological gene-regulatory networks. scGAN also captured gene count distributions and correlations well, and the training time was proportional to the complexity and size of scRNAseq datasets.

Marouf et al. (99) proposed a conditional scGAN model (cscGAN) for the realistic generation of single-cell RNA-seq data. The projection discriminator, along with the Conditional Batch Normalization and LSN function in the generator, is used to generate specific cell types of interest while learning multi-cell type complex data. Conditional generation of cell types could be used to augment the number of sparse specific cell populations that might represent only a small part of the total cells sequenced. However, it may help to solve the class imbalance problem. Similar to the above research, Wang et al. used CGAN for inferring target gene expression profiles by incorporating both adversarial and L1-norm loss terms. Comparative analysis showed that this model outperformed previous linear methods in gene expression inference (104).

Xu et al. proposed the GAN for scRNA-seq imputation (scIGANs), which uses generated realistic rather than observed cells to avoid the limitations, such as many sources of technical noises and dropouts, and the powerless for rare cells. ScIGANs converts the expression profiles of individual cells to images and feeds them to GAN. The trained generative network produces expression profiles representing the realistic cells of defined types. The generated cells, rather than the observed cells, are then used to impute the dropouts of the real cells (6).

Hi-C is commonly used to study three-dimensional genome organization. Hong et al. (105) developed a GAN, namely DeepHiC, to predict high-resolution Hi-C contact maps from low-coverage sequencing data. DeepHiC can reproduce high-resolution Hi-C data from as few as 1% down sampled reads. Application of DeepHiC to Hi-C data on mouse embryonic development can facilitate chromatin loop detection with higher accuracy.

Killoran et al. (102) developed a WGAN-based deep generative network for creating new DNA sequences by encoding the discrete sequences of characters (the nucleotides A, C, G, T) into a continuous representation using one-hot encodings. The authors proposed a joint approach that extends an activation maximization version by incorporating a trained generator model on a dataset of 4.6M 50-nucleotide-long sequences encompassing chromosome 1 of the human genome hg38. This approach is suitable for discrete sequences such as DNA. They found that the generative model can learn important structures from DNA sequences, and can be used to explore and design new DNA sequences with desired properties (102).

Guptaand Zou (103) proposed a novel feedback-loop architecture, called Feedback GAN (FBGAN) to optimize synthetic gene sequences for desired properties using an external function analyzer. The feedback-loop model consists of two components, including GAN and a differentiable neural network. GAN was used to generate novel raw gene sequences. The differentiable neural network named analyzer converted a gene sequence into a probability that the sequence encoded an antimicrobial peptide (AMP). The *n* top-ranked favorable generated sequences replaced the oldest *n* genes present in the discriminator training dataset. This model was able to generate synthetic genes coding for peptides of up to 50 amino acids in length, and the peptides can be optimized for the secondary alpha-helical structure of the resulting peptides (103).

Anand and Huang (101) applied GAN to generate protein structures by encoding protein structures in terms of pairwise distances between α-carbons on the protein backbone by using data from the Protein Data Bank, and used the Alternating Direction Method of Multipliers (ADMM) and Rosetta algorithm to transform 2D pairwise distances into 3D Cartesian coordinates. The authors compared their work with traditional HMMs-based methods. They found that their generator model could learn to construct meaningful secondary structure elements such as alpha helices and beta sheets. The generated maps were highly variable and similar but not identical to the actual data, indicating that the GAN model was not just memorizing the training data. Finally, the authors verified that the generative model can reconstruct missing sections of corrupted protein structures (101).

Similarly, Yeh et al. (106) also proposed a 2D distance map representation of protein GAN model to predict particular missing regions in a protein structure using the idea of image inpainting. The author used this model to learn the distribution of a particular loop region with the context of the loop region from the candidate patch pool and successfully predicted the loop region (100). Compared to the traditional time-consuming and expensive experimental methods such as X-ray crystallography or Nuclear Magnetic Resonance (NMR), this GAN model is more convenient and time-saving.

Accurate identification of prognostic biomarkers is an important but challenging goal in bioinformatics. Kim et al. (107) applied GAN model to specify candidate prognostic gene module by graph learning algorithms and evaluated genes scores via a PageRank algorithm using multiple-omics data, including copy number, gene expression, DNA methylation, and somatic mutation data from five cancer types. Firstly, they reconstructed functional interaction networks (FIs network) that included known pathways in human biology. Then the reconstructed FIs network was learned by GAN to select features via PageRank with GAN weights, and finally, the prognosis was predicted. They successfully identified a number of genes involved in cancer development and analyzed their roles in biological pathways. Their model showed better predictive accuracy than existing methods.

There are some other applications in bioinformatics using GAN to enhance gene expression classification. Huynh et al. realized new data generation from original training datasets through the combination of GAN with nonlinear Support Vector Machines (SVMs). The results of GAN-SVM model displayed a better performance than the most advanced classification methods, including *k*-nearest neighbors (KNN), SVMs, decision tree (DTs) of C4.5, and random-forest (RFs) (108). Bhat et al. proposed a deep generative machine learning architecture (DeepCancer) to test the ability of GAN in classifying breast cancer and prostate cancer samples via the features learned by the discriminator. The results showed that the generative model achieved a high accuracy score (109).

## Conclusion

In this article, we briefly introduced the origin, working principle, the development history of GAN, and numerous applications to the areas of digital image processing, medical imaging analysis, medical informatics, and bioinformatics.

In digital image processing, GAN can do image generation, high-resolution images generation from low-resolution images and image inpainting, which perform well and are widely used. Considering too many applications in digital image processing, due to space limitations, we only selected the articles with the most citations as the lead application to introduce, which leads to some applications in the directions of image processing weren't introduced comprehensively such as Style migration, image coloring, etc.

From the application of GAN in medical images, we can see that GAN-based models provide a good solution for data shortage in medical image analysis. It can be regarded as one of the important additions to the manual labeling from radiologists. The models based on a single GAN are more used as data augmentation methods to increase the variety and quantity of images in the same modality. On this basis, Cycle-GAN-based models make translations between multiple modalities possible. Involving in segmentation networks within the Cycle-GAN models, cross-modality segmentations can be learned in an end-to-end manner. This will effectively promote the application and development of deep learning algorithms in medical image analysis. However, GAN or Cycle-GAN still have limitations. For example, the CT images from the head cannot be generated from the CT images of the abdomen or MRI images from the legs. Researchers need to carefully design their data flow and lost functions to avoid problems such as non-convergence or model collapse. Additionally, some medical image analysis tasks require detailed 3D information of the organ. Involving 3D feature learning or 3D segmentation in the GAN-based model would be a challenge.

Based on EMR or EHR data, although GAN can generate realistic synthesized discrete data, continuous data, and even time series data to solve the issues of fewer labels and unbalanced classifications in medical informatics, there are still some limitations. How to evaluate these generated data and how to apply these generated data to solve medical problems has been controversial, which requires real data to validate.

So far, the application of GAN models in bioinformatics is still in a relatively early stage of development. Most studies applied GAN to generate and/or augment datasets. The above results have demonstrated the similarity of the data generated using GAN models to the original data. Most machine learning algorithms work well when the number of cases in each class is roughly equal. So, using the generated data, we can not only perform a lot of downstream analyses, such as detecting marker genes, dimensionality reduction and clustering, and reconstructing a particular secondary structure, but also decrease the number of human and animal experiments with a concomitant reduction in experimental costs, addressing important ethical and financial issues. In addition, GAN framework can work with any type of neural networks, so its application in bioinformatics will be more extensive.

In conclusion, more and more applications of GAN in biomedical research are being proposed. Some of GANs such as WGAN, Cycle-GAN, CGAN, medGAN are receiving more and more attention because of their importance in biomedical research. Although GAN has its advantages in simulating various problems, there are also some limitations. For example, when the sample size is small, the accuracy of the model will be relatively low. As GAN uses deep neural networks as generators, poor interpretability is also a common problem.

## Author Contributions

LL, LY, ZZ, and ZF reviewed the literature and wrote the initial manuscript. LL, NZ, and XZ proposed an article outline. WZ, XZ, NZ, and YC revised the drafts. LL created and maintained an EndNote database.

## Conflict of Interest

The authors declare that the research was conducted in the absence of any commercial or financial relationships that could be construed as a potential conflict of interest.
